# The Prevalence of Asthma and Asthma-Like Symptoms among Seasonal Agricultural Workers

**DOI:** 10.1155/2020/3495272

**Published:** 2020-06-25

**Authors:** Yasemin Saglan, Ugur Bilge, Dilek Oztas, Ramazan Saglan, Yunus Emre Sarı, Huseyin Balcıoglu, Ilhami Unluoglu

**Affiliations:** ^1^Eskisehir Provincial Directorate of Health, Eskisehir, Turkey; ^2^Department of Family Medicine, Faculty of Medicine, Eskisehir Osmangazi University, Eskisehir, Turkey; ^3^Department of Public Health, Faculty of Medicine, Ankara Yıldırım Beyazıt University, Ankara, Turkey; ^4^Cal State Hospital, Denizli, Turkey

## Abstract

**Aim:**

The aim of the study was to determine the prevalence and risk factors of asthma and asthma-like symptoms in seasonal agricultural workers living in fields with toxic chemical exposure.

**Methods:**

European Community Respiratory Health Survey (ECRHS) questionnaire was used to assess the prevalence of asthma and asthma-like symptoms in the study.

**Results:**

Of the study group, 51.1% (267) were male and the age of the study group ranged from 18 to 88 years and the mean (SD) was 45.68 (13.39) years. The prevalence of asthma attacks in seasonal agricultural workers in the last one year (current prevalence) was 11.2%; the prevalence of asthma (cumulative prevalence) was 15.1%. In the study, smoking was found to be an important risk factor for current asthma. The prevalence of cumulative asthma was higher in seasonal agricultural workers with allergic rhinitis (*p* < 0.05 for each).

**Conclusions:**

Seasonal agricultural workers are exposed to the worst conditions of working groups. These difficult conditions also cause many health problems. Asthma has also been identified as an important health problem among seasonal agricultural workers.

## 1. Introduction

Seasonal agricultural workers are agricultural workers migrating to places where agricultural demand is high, migrating to their own countries at the end of the season. This group is a vulnerable group because of the inadequacy of living conditions and the inability to reach basic human rights services [1]. The main causes of health problems of agricultural workers can be summarized as education level, low socioeconomic level, bad climatic conditions, safety of working tools, working hours, direct contact with animals and plants, bite, poisoning, parasites, allergies, and chemical products used [2]. Toxic chemicals such as pesticides may contribute to airway reactivity and asthma among agricultural workers. Chemicals may cause exacerbation of asthma. Pesticides may also modulate inflammatory responses to other agricultural exposures, such as endotoxin and allergens [3, 4]. Many temporary or permanent health problems can occur in this group [5].

The main health problems of agricultural workers can be grouped as respiratory diseases, musculoskeletal diseases, infectious diseases, accidents and injuries, dermatological diseases, and psychosocial problems [6]. Seasonal agricultural workers are recognized as a population requiring special attention by the health community. This designation as a “special population” is associated with higher than average occupational risk exposures as well as poorer than average health status [1].

Agricultural workers have been shown to have increased especially rates of respiratory symptoms and diseases due to respiratory irritants, toxic chemicals, and allergens [7]. Respiratory disease is a major health risk for those working in agriculture. The most common cause of asthma among agricultural workers is a result of exposure to agricultural dust and chemicals. When both are inhaled, they can trigger an allergic reaction in the respiratory system [8]. These problems usually become chronic because of the lack of access to healthcare and repeated exposures [9].

Asthma is a chronic inflammatory disease of the respiratory tract characterized by unstable obstruction of the airflow and severe response of the airways [10]. Asthma is a multifactorial disease in which family, as well as infectious, allergic, socioeconomic, psychological, and environmental factors, play a role [11]. The characteristics of asthma include recurring symptoms, reversible airflow obstruction, and bronchospasm. The symptoms of this disease include wheezing, coughing, chest tightness, and shortness of breath [12].

The aim of the study was to determine the prevalence and risk factors of asthma and asthma-like symptoms in seasonal agricultural workers living in fields with toxic chemical exposure.

## 2. Material and Method

The study is a cross-sectional study that was conducted on seasonal agricultural workers working in the rural area of Eskişehir (Turkey) in 2017. The questionnaire consists of a sociodemographic questionnaire and European Community Respiratory Health Survey (ECRHS) questionnaire.

ECRHS questionnaire was used to assess the prevalence of asthma and asthma-like symptoms in the study [13, 14]. Based on the ECRHS questionnaire, current asthma is defined as recently taking antiasthma medication or having an asthma attack in the last 12 months, and cumulative asthma is defined as having an attack of asthma at any time in life.

The ECRHS questionnaire includes asthma symptoms: wheezing in the past 12 months, wheezing with shortness of breath in the last 12 months, wheezing in the absence of cold during the past 12 months, feeling tightness in the chest while waking up in the past 12 months, nocturnal cough and dyspnea attacks in the last 12 months, a history of asthma attack in the last 12 months, recent antiasthma medication, and nasal allergies [15].

### 2.1. Study Sample

In the study, the prevalence of asthma was assumed to be 5% [16–18], the margin of error was taken as 2%, the confidence interval was taken as 95%, and the sample size was calculated at least as 456. A total of 560 seasonal agricultural workers were approached, but only 523 of them agreed to participate. The response rate was 93.3%.

### 2.2. Ethical Procedure

The approval of Socıal and Human Scıences Ethıcs Committee of Ankara Yıldırım Beyazıt Unıversıty was confirmed.

### 2.3. Data Analysis Plan

Statistical Package for Social Sciences (SPSS 24.0) was used to evaluate the obtained data on the computer. Chi-squared test was used for univariate analysis, and multivariate logistic regression analysis was used with entering method in multivariate analysis. Results were evaluated at a 95% confidence interval, and *p* ≤ 0.05 was accepted as significant for all variables.

## 3. Results

Of the study group, 51.1% (267) were male and the age of the study group ranged from 18 to 88 years and the mean (SD) was 45.68 (13.39) years. The prevalence of asthma attacks in seasonal agricultural workers in the last one year (current prevalence) was 11.2%; the prevalence of asthma (cumulative prevalence) was 15.1%. In the study, the prevalence of allergic rhinitis was 23.5% and the prevalence of allergic rhinitis was higher in females than in males (*p* = 0.034) in seasonal agricultural workers.

The prevalence of wheezing was higher in males than in females (*p* = 0.042). No correlation was found between gender and wheezing with breathlessness, wheezing without cold, woken up with chest tightness, woken up with shortness of breath, woken up with cough, current asthma, and cumulative asthma among seasonal agricultural workers.

The distribution of asthma and asthma-like symptoms according to gender in univariate models is given in Table 1.

In the study, the presence of family histories of atopy was found to be an important risk factor for wheezing with breathlessness and woken up with chest tightness among seasonal agricultural workers (*p* < 0.05).

In patients with allergic rhinitis, the prevalence of woken up with shortness of breath was 1.527 times higher than those without allergic rhinitis (*p* < 0.05). Smoking was found to be an important risk factor for current asthma (*p* < 0.05). The prevalence of cumulative asthma was higher in seasonal agricultural workers with allergic rhinitis (*p* < 0.05).

Gender, smoking status, pruritus dermatitis and/or eczema, allergic rhinitis diagnosis, and family history of atopy were evaluated as risk factors, and the distribution of asthma and asthma-like symptoms according to risk factors in multivariate models is given in Table 2.

## 4. Discussion

Allergic diseases, including asthma, rhinitis, and eczema, have emerged as a global public health challenge due to their elevated prevalence [19].

Asthma is a chronic inflammatory disease of the airways that affects approximately 300 million people in the world and 3.5 million people in our country [20]. Agricultural workers are at increased risk for respiratory diseases, including asthma, as a result of exposure to chemicals, grains, animals, and dusts and other agricultural exposures [21], and this population is exposed to bad conditions within the working groups [22, 23]. Pesticides may contribute to asthma among agricultural workers [24].

Differences in living conditions, air and other types of environmental pollution, smoking, and genetic factors may contribute to discrepancy [19]. In a case report made by Çımrın and Karaman, it was found that seasonal agricultural worker developed asthma due to dust exposure [25].

The prevalence of asthma attacks in seasonal agricultural workers in the last one year (current prevalence) was 11.2%; the prevalence of asthma (cumulative prevalence) was 15.1% in our study. The prevalence of asthma varies according to geographical regions.

In another study conducted in Eskisehir, the prevalence of current asthma, cumulative asthma, and allergic rhinitis was 5.9%, 5.9%, and 37.6%, respectively [26]. In our study in Eskisehir, the prevalence of current asthma, cumulative asthma, and allergic rhinitis was 11.2%, 15.1%, and 23.5%, respectively. We think that the rate of asthma in seasonal agricultural workers is higher than that in the general population, which may be caused by toxic chemicals and environmental exposure.

The prevalence of asthma in different countries has been reported in the range of 2.4-18.4% [27, 28]. In a cross-sectional prevalence study conducted in Iran in 2015-2016 with ECRHS survey, the prevalence of asthma in adults was 8.9% [29]. Similar to our study, in a Swedish study conducted in 2008, the prevalence of asthma was found to be 11.8% [30].

In our study, prevalence of wheezing was higher in males than in females (*p* = 0.042). In a study conducted with the ECRHS survey in Saudi Arabia in 2016, the prevalence of wheezing in the last 12 months was 18.2% and the difference between men and women was not significant (*p* = 0.107) [31].

In the study, the presence of family histories of atopy was found to be an important risk factor for wheezing with breathlessness and woken up with chest tightness among seasonal agricultural workers (*p* < 0.05). In a study by Wang et al., the prevalence of asthma was found to be 3.15 times higher in participants with a history of allergic diseases in the mother than in nonallergic mothers [32]. In another study, if one of the parents has asthma, the risk of asthma in the child increases to 20-30%, and if both parents have asthma, this risk increases to 60-70% [29].

The prevalence of allergic rhinitis was determined as 23.5%, and allergic rhinitis was significantly higher in seasonal agricultural workers in our study (*p* < 0.05). In a study by Yorgancıoğlu et al., the prevalence of allergic rhinitis in our country varies between 8.9% and 27.7% [33]. Increasing prevalence of allergic rhinitis, changing living conditions, environmental and air pollution, childhood infections, longer indoor life, smoking, changes in dietary habits, and some genetic factors are responsible [34]. Allergic rhinitis was found to be 28.3% in a study conducted in Tehran between 2013 and 2016 similarly [35].

In our study, the prevalence of allergic rhinitis was higher in females than in males (*p* = 0.034) in seasonal agricultural workers. Similarly in a study by Nihlén et al., it was reported that allergic rhinitis was more common in women, and the authors stated that the reason for this was that women may be more inclined to write allergy symptoms in the questionnaire [36].

Smoking was found to be an important risk factor for current asthma in our study (*p* < 0.05). In a study by Idani et al., the prevalence of current asthma, asthma symptoms, wheezing, nocturnal cough, and asthma medication was significantly higher in smokers than nonsmokers [37]. In a study consistent with our study, smoking and/or smoke exposure have been shown to lead to exacerbation of lung function in asthmatics, asthma symptoms, and weight gain [38].

Strengths of the study are that the number of cases is higher than the number of samples and the number of women and men is approximate (51.1/48.9).

The limitations of the study are that functional lung monitoring (spirometry, test, PEF monitoring, etc.) procedures that will support the diagnosis of asthma cannot be applied, the study is carried out only in the Eskisehir region, and workers under the age of 18 are not included.

## 5. Conclusions

Seasonal agricultural workers are one of the occupational groups that are exposed to bad conditions within the working groups. In this group, health problems are frequently encountered throughout the world. Allergic respiratory diseases have also been identified as an important health problem among seasonal agricultural workers. It should not be forgotten that seasonal agricultural workers who apply to the outpatient clinic may have asthma-like symptoms and allergic conditions. Similarly, it should be questioned what the occupation of these patients is in these symptoms, and it should be known that seasonal agricultural workers may have these symptoms. It is estimated that allergic respiratory diseases are highly observed in seasonal agricultural workers due to environmental factors. So respiratory diseases which can be due to toxic chemicals and environmental exposures should be investigated in seasonal agricultural workers.

## Figures and Tables

**Table 1:** The distribution of asthma and asthma-like symptoms according to gender in univariate models.

Asthma and asthma-like symptoms	Gender		Total	OR (CI 95%)^∗^	*p*
	Male, *n* (%)	Female, *n* (%)			
Wheezing	23 (8.6)	20 (7.8)	43 (8.2)	1.269 (1.090-1.789)	0.042^∗∗^
Wheezing with breathlessness	5 (21.7)	4 (20.0)	9 (20.9)	1.197 (0.734-1.954)	0.471
Wheezing without cold	8 (34.7)	7 (35.0)	15 (34.8)	0.756 (0.463-1.234)	0.263
Woken up with chest tightness	28 (10.4)	23 (8.9)	51 (9.7)	0.854 (0.606-1.203)	0.366
Woken up with shortness of breath	27 (10.1)	21 (8.2)	48 (9.1)	1.151 (0.816-1.624)	0.422
Woken up with cough	36 (13.4)	32 (12.5)	68 (13.0)	0.800 (0.567-1.128)	0.800
Allergic rhinitis	60 (22.5)	63 (24.6)	123 (23.5)	1.312 (1.083-1.812)	0.034^∗∗^
Current asthma	33 (12.3)	26 (10.1)	59 (11.2)	1.146 (0.776-1.691)	0.493
Cumulative asthma	42 (15.7)	37 (14.4)	79 (15.1)	1.108 (0.786-1.562)	0.558

^∗^OR: odds ratio; CI: confidence interval. ^∗∗^*p* < 0.05.

**Table 2:** The distribution of asthma and asthma-like symptoms according to risk factors in multivariate models.

Asthma and asthma-like symptoms	Gender OR (CI 95%)^∗^	Smoking OR (CI 95%)^∗^	Itching dermatitis and/or eczema OR (CI 95%)^∗^	Allergic rhinitis OR (CI 95%)^∗^	Family histories of atopy OR (CI 95%)^∗^
Wheezing	1.238 (0.875-1.753)	0.884 (0.625-1.250)	1.222 (0.865-1.726)	0.853 (0.603-1.205)	0.922 (0.652-1.352)
Wheezing with breathlessness	1.253 (0.758-2.071)	0.995 (0.603-1.641)	1.252 (0.762-2.057)	0.809 (0.492-1.331)	1.861 (1.129-3.069)^∗∗^
Wheezing without cold	0.785 (0.476-1.293)	0.764 (0.464-1.259)	0.878 (0.535-1.440)	1.517 (0.925-2.489)	0.875 (0.532-1.437)
Woken up with chest tightness	0.819 (0.578-1.162)	0.761 (0.537-1.078)	1.181 (0.835-1.672)	1.115 (0.788-1.578)	1.442 (1.018-2.041)^∗∗^
Woken up with shortness of breath	1.125 (0.793-1.596)	1.133 (0.800-1.606)	1.067 (0.754-1.511)	1.527 (1.079-2.161)^∗∗^	0.839 (0.593-1.188)
Woken up with cough	0.791 (0.559-1.120)	1.028 (0.728-1.452)	1.129 (0.799-1.595)	1.056 (0.747-1.491)	1.060 (0.751-1.497)
Current asthma	1.126 (0.758-1.672)	1.446 (1.071-2.154)^∗∗^	0.921 (0.622-1.365)	0.796 (0.537-1.180)	1.152 (0.777-1.707)
Cumulative asthma	1.143 (0.807-1.620)	1.049 (0.742-1.484)	1.015 (0.718-1.435)	1.495 (1.057-2.114)^∗∗^	1.030 (0.729-1.457)

^∗^OR: odds ratio; CI: confidence interval. ^∗∗^*p* < 0.05.

## Data Availability

The data used to support the findings of this study are available from the corresponding author upon request.
